# Supplementary material for: Patterns of Intron Gain and Loss in Fungi
Source: PLoS Biol. 2004 Nov 30;2(12):e422. doi: 10.1371/journal.pbio.0020422 (PMC532390; doi:10.1371/journal.pbio.0020422)
Supplement: Table S1 — Also available at http://genes.mit.edu/NielsenEtAl/. (4.3 MB ZIP). [file pbio.0020422.st001.zip › NielsenEtAl/html/977.html]

AN1379.1.NCU04288.1.MG00946.1.FG10280.1


```
 CLUSTAL W (1.82) Multiple Sequence Alignments - Introns Inserted


Sequence 1: MG00946.1	358 aa
Sequence 2: FG10280.1	356 aa
Sequence 3: NCU04288.1	357 aa
Sequence 4: AN1379.1	362 aa
Alignment Length: 366 aa
Number Identitical Residues: 192 aa
Alignment Score (without introns) 9865


MG00946.1 	MSGLFGG--GAAAAQPNTVGDLKNDVAIANPPEDSISDLAFNPNQADQKDFLAVSSWDKK
NCU04288.1	MAGLFG---SAAASASNTLGDLKQDVELGQPPEDSISDLAFNPNPADQKDFLAVASWDKK
FG10280.1 	MSTLFG---SASSAAATSIGDLKQDVALSDPPTDTISGLSFSP-APNGPDFLAISSWDNK
AN1379.1  	MSGLFGATANTSTAASNTTGDISKDVALNSPPEDSISDLRFSP----ASEHLAVASWDKK
          	*: ***.::.:::: ..: **:.:** : .** *:**.* *.*      :.**::***:*

MG00946.1 	VRIYEVLGNGTAEGKHAYEHDGPVFSVDYYK0DGTKIVSGGADKQAKVCDMNT--GQTAQ
NCU04288.1	TRIYEILSNGQGQGQAMIEHDAPVFSCDFFK0DGTKVISAGADKAAKVLDLAT--GQSMQ
FG10280.1 	VRIYEIATNGQSQGRHAYEHSQPVLSCDFSK0DGTKIVSAGADKNIKVCDLAS--QQDIV
AN1379.1  	VRIYEINEQGQSEGKALFEHEAPVLNCCWSP0DGTKVVGAGADKAARMLDLAANATAPIQ
          	.****:  :* .:*:   **. **:.  :   ****::..****  :: *: :.:     

MG00946.1 	VAQHEKPVRSVRWFD--NNGSPMVITGSWDKTVKYWDLRQQSPVATLQCQERVYTMDVRD
NCU04288.1	VAAHDMPIKCVRYFE--ANGTPMAVTGGWDKQIKYWDFRSANPAATVQAQERVYTMDVRD
FG10280.1 	VGTHDQPVRTARFFD--SGNGPMVVSGSWDKTVKYWDLRQQGPAATVACQERVYTMDVRD
AN1379.1  	VAAHDAPIRCCHMIPNPAGGTPLLVTGSWDKQVKYWDLRQSTPIASLECQERVYTMDVKD
          	*. *: *::  : : .. .. *: ::*.*** :****:*.  * *:: .*********:*

MG00946.1 	KLLVIGTADRYINVVNLNEPQKFYKTLQSPLKWQTRVVSCFTDANGFAIGSIEGRCAIQY
NCU04288.1	NLLVVGTADRYINVINLKDPGKFYKTMQSPLKWQTRVVSCFNDSQGFAIGSIEGRCAIQY
FG10280.1 	NLCVVGTADRYINVIDLKNPTKFYKTLQSPLKWQTRVVSCFTDSAGFAIGSIEGRCAIQY
AN1379.1  	KLLVIGTADRYINIVDLNNPTKFYKTMQSPLKWQTRVVSCFTDANGFAVGSIEGRCAIQY
          	:* *:********:::*::* *****:**************.*: ***:***********

MG00946.1 	VEDKDSS2SNFSFKCHRDAP--QGSVTSVHAVNDISFHPVHGTFSTAGSDGTFHFWDKDA
NCU04288.1	VEDKDSA2SNFSFKCHRDPA--QGNTTAVHAVNDISFHPQHGTFSTAGSDGTFHFWDKDA
FG10280.1 	VEAKDSS2ANFSFKCHRDPP--ANGVTNVHAVNDISFHPVHGTFSTAGSDGTFHFWDKDA
AN1379.1  	VEDKDSS~SNFSFKCHRETPPNQRDINNIYSVNAISFHPVHGTFSTAGADGTFHFWDKDA
          	** ***: :********:....  . . :::** ***** ********:***********

MG00946.1 	KHRLKGYPNVGGSITATTFNKNGNIFAYAVSYDWAKGYQGNNSTYPNKVMLHPVQQDECK
NCU04288.1	KHRLKGYPNVGGSITSTTFNKTGSIFAYAISYDWSKGYQGNSPTYPTKVMLHPVQQDECK
FG10280.1 	KHRLKGYPNVGGSITSTTFNKNGSIFAYAVGYDWAKGYQHNTQSYPIKVMLHPVTNDECK
AN1379.1  	KHRLKGYPSVGGTISSTAFNRTGNIFAYAVSYDWSKGYSANTPQLPNKVMLHPVAQEEVK
          	********.***:*::*:**:.*.*****:.***:***. *.   * ******* ::* *

MG00946.1 	PRPSVKKR
NCU04288.1	PRPSVKKR
FG10280.1 	PRPSAKKR
AN1379.1  	PRQNARRR
          	** ..::*
```
